# *“Moving like birds”*: A qualitative study of population mobility and health implications in the Bijagós Islands, Guinea Bissau

**DOI:** 10.1016/j.socscimed.2019.03.019

**Published:** 2019-06

**Authors:** Sophie Durrans, Anna Last, Hamadou Boiro, Adriana Goncalves, David Mabey, Katie Greenland

**Affiliations:** a Department for Disease Control, London School of Hygiene and Tropical Medicine, Keppel Street, WC1E 7HT, London, UK; b Department of Clinical Research, London School of Hygiene and Tropical Medicine, Keppel Street, WC1E 7HT, London, UK; c National Institute for Studies and Research, Bissau, Guinea-Bissau and Faculty of Sociology, Anthropology and Folkloristics, University of Iceland, Reykjavik, Iceland

**Keywords:** Guinea-Bissau, Population mobility, Disease transmission, Disease control

## Abstract

Population movement is a major driver for infectious disease transmission and can impact the success of disease control and elimination strategies. The relationship between disease transmission and permanent migration is well documented, but fewer studies have considered how different types of population mobility affects disease transmission and control programmes.

This qualitative study was conducted on two islands of the Bijagós archipelago, Guinea Bissau to understand spatial and temporal population movement, and reasons for these movements, within, between and away from the Bijagós islands. Data were collected on two islands using key informant interviews (n = 8), daily activity-location interviews (n = 30) and focus group discussions (n = 6). Data were analysed thematically using an adapted typology of mobility.

Findings revealed that movement within and between islands, and from islands to the mainland, was a common feature of island life for men and women alike. It was usual for trips away from home to last for several months at a time. Five key reasons for travel were identified: subsistence activities; family events; income generating activities; cultural festivities and healthcare. These movements often occurred erratically all year round, with the exception of seasonal travel within and between islands for agricultural purposes.

Our study characterised detailed patterns of human mobility in the Bijagós islands as a first step towards understanding the potential impact of different types of mobility on disease exposure, transmission and public health programmes. Short-term mobility may have a significant impact on the spread of infectious diseases with short incubation periods. Predictable movements, such as travel for seasonal agricultural work, should be taken into account for tailoring and increasing the reach of public health interventions. Further research is needed to understand the role of human behaviour and mobility in disease transmission and control across the archipelago.

## Highlights

• Circulatory movement of people affects public health differently to migration.
• Uses mobility typology to categorise movement across the Bijagós Islands.
• Demographic and cultural factors play a significant role in understanding mobility.
• Consider types of mobility to improve public health programmes.

## 1 Introduction

### 1.1 Population mobility and public health

Population movement is a major driver for infectious disease transmission and can impact the success of disease control and elimination strategies. The public health challenges associated with population movements are widely documented (Gushulak and Macpherson, 2004b). Migration (mobility involving a permanent change of residence) is most commonly studied in relation to the effect of population movement on disease (Sattenspiel and Powell, 1993). Migration can change the spatial spread of infectious disease and international migration often affects the health system of both the origin and host country (Gushulak and MacPherson, 2006). However, many types of population movement exist. These include (but are not limited to): displaced populations (due to disaster or conflict); labourers; emigrants and immigrants; visitors; education travel; and circulatory movement, which is temporary travel “away from a recognised place of residence but with eventual return” (Gould and Prothero, 1975). For each type of travel, the process of mobility has three phases relating to health: premovement, the journey itself, and arrival (Gushulak and Macpherson, 2004a). Factors that can influence health during each phase include the incidence and prevalence of diseases, as well as social, cultural and environmental determinants.

Population movement has been explored in multiple contexts in sub-Saharan Africa (HOSEGOOD et al., 2005, Lagarde et al., 2003; Bruijn et al., 2001; Prothero, 1977). Local, regional and international population mobility can play a significant role in the spatial spread of disease. This was the case in the Ebola outbreak in West Africa from 2014 to 2016 (Alexander et al., 2015), which resulted in 28,000 cases and 11,000 deaths (WHO, 2016). Circulation is characteristic of West African populations (Awumbila et al., 2014), but some suggest that movement has changed considerably in recent years and efforts must be made to understand mobility patterns (Halloran et al., 2014). While quantitative methods can provide insight into the time and place of movements, qualitative methods can help to understand the social processes that shape population movement (Kloos et al., 2010, Watts et al., 1998).

Few studies have aimed to understand how population mobility can impact health within island settings. Approximately 600 million people – 10% of the world's population - live on islands (KING, 2009). From a biological perspective, islands are ideal sites to study biodiversity and the epidemiology of infectious diseases (Acevedo et al., 2013). From a cultural perspective, human mobility is unambiguously associated with island livelihoods (Christensen and Mertz, 2010). Small island populations can face a range of health issues which climate change can exacerbate, due to changes in disease prevalence or forced population movement (Singh et al., 2001; Mcmichael et al., 2006). Understanding the patterns and processes of human mobility in island settings may therefore inform programmes and policies to improve health, and could potentially be a model to investigate diseases in similar settings.

### 1.2 Typology of mobility

This paper draws on a descriptive typology of mobility in Africa which has been influential in the field of mobility and health (Prothero, 1977). The typology categorises types of population movement according to time and space (Gould and Prothero, 1975). While circulation acknowledges movement away with eventual return, migration assumes permanent movement. Prothero argued that the distinction between migration and circulation has been overlooked, as circulation can have different impacts on health. He identified four types of circulatory movement: *daily* (away from place of residence for up to 24 h); *periodic* (away for more than 24 h and less than 12 months); *seasonal* (a category of periodic, where the time absent relates to a season); and *long-term* (lasting more than 12 months). Movement was also classified according to four spatial pathways in sub-Saharan Africa: rural-rural, rural-urban, urban-rural and urban-urban.

This typology demonstrates the impact that some types of population movement have on exposure to different health hazards. Moving from one ecological zone to another could increase exposure to diseases. Gushulak and MacPherson similarly argue that disparity “is perhaps the most important factor in the relationship between mobility and health”. This can involve travel between disparate risk environments, or between disparate health systems (Gushulak and Macpherson, 2004a). Movements that involve contact with new groups of people - either during the journey or on arrival - could lead to possible transmission of infectious diseases. The typology also highlights that physical factors (e.g. fatigue and malnutrition) and psychological factors (e.g. psychosocial stress) can impact health.

### 1.3 The Bijagós archipelago

The Bijagós archipelago is a collection of 88 islands off the coast of Guinea-Bissau, West Africa. Only sixteen of these islands are permanently inhabited with a total population of around 24,000 (Instituto Nacional De Estatística, 2010). Many of the uninhabited islands are reserved for seasonal agricultural use, domestic livestock and sacred ceremonies (Cross, 2014). The islands are culturally and ecologically complex. Bijagós society is matrilineal with matriarchal elements (Cross, 2015, Thompson et al., 2015) and the islands host a diversity of ecosystems from mangroves to forests.

The islands are co-endemic for malaria and Neglected Tropical Diseases (NTDs) such as lymphatic filariasis, scabies and soil transmitted helminths (Thompson et al., 2015; Marsden et al., 2013, Last et al., 2014). Trachoma is close to elimination from the islands and is currently under surveillance (Last et al., 2017). Whilst the Guinea-Bissau Ministry of Health has prioritised NTD elimination, there is a lack of good quality, recent epidemiological, social and behavioural data on the burden of these diseases and their local transmission patterns. The authors of this paper are currently undertaking an integrated mapping of malaria and NTDs to gain insight into their patterns of transmission through social, behavioural, epidemiological and vector studies.

Little research has been conducted that details mobility patterns and reasons for movement on the archipelago. Several researchers have suggested that there is a high level of mobility within and between the islands due to the slash-and-burn agriculture style (Klute and Fernandes, 2014) and secret sites for age-related initiation ceremonies (Cross, 2014), although long distance mobility is assumed to be less common. The majority of the population is indigenous, although small numbers of people have migrated from other West African countries to the islands to work in small scale fisheries (Cross, 2015) or for trade in the very small town in Bubaque, the only ‘urban centre’ (Bordonaro, 2009). It is plausible that population mobility could impact disease transmission dynamics and the success of disease control and elimination strategies on the islands. One study has made a link between population mobility and malaria, suggesting that mosquitoes accompanied people in high volume movement via local boats between the mainland and the islands, and amongst the islands themselves (Marsden et al., 2013). Healthcare provision on the islands is largely based around public health campaigns such as vaccination and community mass drug administration strategies. As population coverage is crucial to success, movement can be a barrier to disease control or elimination efforts.

This study is a first step towards exploring population mobility within islands, between islands, and between islands and the mainland, in order to identify opportunities for further research on population movement, behavioural risk factors and control and elimination strategies for infectious diseases. The paper adapts a typology first developed by Prothero (1977) to outline temporal and spatial dimensions of movements and associated health hazards. The study aims to answer the following research questions: How do social and demographic factors (including age, gender and family structure) impact population movement? What type of movements occur and why, and how frequent are they? These questions will inform a discussion on the potential health implications of types of mobility on the Bijagós Islands.

## 2 Methods

### 2.1 Study design and setting

Based on an ethnographic epistemology, this study aims to understand the type of population movement and reasons for movement within and between islands, and between the Bijagós archipelago and the mainland. Data were collected over three weeks in November 2017 across seven sites on two islands; Bubaque and Canhabaque. Data collection methods consisted of in-depth interviews and focus group discussions (FGDs).

Whilst the majority of the islands in the archipelago are relatively homogenous in terms of population and activities, Bubaque is the only island with a small centre which hosts a population of approximately 4000 people (Instituto Nacional De Estatística, 2010). This is the site of the limited hospital, market, shops, small guesthouses, restaurants, education and administrative services, and a radio station with transmission to all islands (Thompson et al., 2015). We selected Bubaque due to its prominence as a transport hub of the archipelago, with regular small ferries and other boats going to the capital, Bissau, twice weekly, in addition to transport to other islands. Ferry journeys between Bissau and Bubaque take approximately 4 h.

Canhabaque is a larger island east of Bubaque and contains 19 rural villages, with a population of 3500 people (Instituto Nacional De Estatística, 2010). Small local boats (*canoa)* travel between Bubaque and Canhabaque approximately twice a week, and journeys can take up to 2 h. There is a small health post staffed by two community nurses on Canhabaque and small shops within some villages selling limited goods. With no roads, infrastructure is very limited. We chose this island as our second site after initial interviews with key informants revealed that travel between Bubaque and Canhabaque was frequent. Canhabaque also has cultural and historical differences that made it an interesting comparison settlement.

### 2.2 Research framework

With the ultimate aim of exploring how population mobility affects disease transmission and behavioural risk factors on the Bijagós Islands, we have adapted Prothero's typology of mobility to fit the archipelago context. We used three spatial categories to reflect the island context: intra-island, inter-island, and movement between an island and the mainland (island-mainland). We also divided the periodic circulation category into short-term (travel from 24 h to one month) and medium-term (travel from 1 to 12 months). Based on the hypothesis that population circulation has a bigger impact on the epidemiology of diseases than permanent migration (Kloos et al., 2010), the adapted framework is used to explore the temporal and spatial aspects of mobility, and how these could have broader implications for infectious disease, based on two of Prothero's health hazards on moving between ecological zones and contact with different people (described in section 1.2). In line with Prothero's original framework, we detail reasons for movement *away* from a place of origin, rather than reasons for return. This revised framework helps to answer the research questions that underpin this study.

### 2.3 Data collection

Data collection was led by a female researcher with training in qualitative methods, and supported by a Guinean qualitative researcher fluent in Kriol (lingua franca), and translators from the islands fluent in the local Bijogo language. In total, we conducted eight in-depth interviews with key informants, 30 daily activity-location interviews with community members and six focus group discussions. These methods will be explained in the following sections. For all methods, we used flexible topic guides with open-ended questions to allow for probing of emergent themes. As the study aims to reflect the range of potential reasons around why, when and where people travel, at each site we purposively sampled to include individuals with a wide range of livelihoods and occupations. For daily activity-location interviews and focus groups, where possible we selected participants who had children of primary school age or younger, since we wanted to understand how age and family structures affect mobility. Voluntary written informed consent was obtained from each participant and all interviews and FGDs were audio-recorded and conducted in either one of two local languages (widely-spoken Kriol or Bijogo) and verbally translated into English. Detailed handwritten notes were taken throughout.

#### 2.3.1 Key informant interviews

Key informants were identified by a local health assistant or field worker familiar with the study site. Six men and two women were selected for interview; one per settlement, except in the town where two informants were chosen due to the relatively large population size. A local assistant selected individuals who they considered a respected elder or held a position of power within their community, such as a village king or *okinka* (queen-priestess), appointed rather than inherited roles of social importance. All key informant interviews were semi-structured, and aimed to understand how population movement is impacted by status, age and societal dynamics. Topic guides included questions on changes to mobility over time within the community as well as personal experiences of movement.

#### 2.3.2 Daily activity-location interviews

We conducted daily activity interviews with 13 men and 17 women at their homes. These interviews aimed to gain insight into the social, economic and gender-related factors that may impact daily movements of individuals and others living in the same household. We asked participants about their hourly actions and movements throughout the previous day, whether they considered this a typical day, and if not, to describe their usual daily activities and whether this varies at different times of the year. We used picture cards to prompt discussion around activities that occur within the home or elsewhere. Cards representing an activity that happens at home or in the immediate surroundings were placed chronologically in one line, while images representing activities that happened away from the home were placed below on a separate horizontal line. This led to the creation of a daily ‘activity-location’ profile for the participant as well as their spouse (if they were aware of their daily routines). These were used to identify when and why people move, and potential health hazards that could lead to different disease exposures, for example coming into contact with different groups of people, or exposure to water sources and different ecologies.

#### 2.3.3 Focus group discussions

Three FGDs were conducted with men, and three with women. Six to eight individuals participated in each FGD. Two FGDs took place in the town, two in villages in Bubaque and two in villages in Canhabaque. We selected participants of similar age who lived in the same village or town district. One group was purposively selected to include older men (aged 57–70) who had completed a traditional age-grade initiation ceremony (*fanado)* specific to the Bijagós. The remaining FGDs were a convenience sample identified with the assistance of a local field worker. All focus groups explored variations in mobility over the course of a year. In each, we asked men or women about their perceptions of movement and activities during different seasons, including short-term and long-term absences from the home. We produced seasonal calendars to visually record information relating to reasons for travel during different months and seasons, and used these as prompts for further discussion.

### 2.4 Data analysis

Data were analysed both visually and temporally to understand the direction and location of movement among the Bijagós islands and between islands and the mainland, and then according to the temporal nature and reasons for these movements. Selected interviews were transcribed and translated from electronic recordings verbatim to English. To protect participants’ anonymity, personal identifiers were not included in transcripts.

#### 2.4.1 Visual analysis of activity patterns

Data from interviews and FGDs was used to create a map (extracted from OpenStreetMap.org and edited in Microsoft Word) detailing inter-island and island-mainland movement away from Bubaque within the last 12 months. For the sake of visual clarity, we only represent movement away from one island, omitting both the time spent away from home and intra-island movements within Bubaque. Arrows indicate the direction and location of movement. The reasons for movement were coded for and categorised by two researchers in two stages, and resulted in five overarching categories outlined in the map's key: subsistence activities (including cultivating rice, collecting cashew or reeds, and fishing); family visits (e.g. weddings, funerals or social visits); income generation activities (e.g. buying and selling goods), cultural festivities (including age-grade initiation ceremonies and football tournaments); and healthcare (e.g. hospital visits or traditional healing).

Data relating to activities performed at particular times of the day were obtained through in-depth interviews, summarised and visually described in two pie charts produced in Microsoft Excel, which detail typical daily activities for men and for women. This is useful to gain understanding of mobility during a typical day, and how mobility differs by gender (Nabasa, 1995). Data from notes and transcripts were aggregated and common activities at similar times were extracted to develop the pie charts. Each pie chart represents a 24 h clock, and splits daily activities according to time. Describing daily mobility patterns is important for understanding disease transmission dynamics. Cairncross et al. (1996) categorises two domains – the public and domestic – as important in the transmission of disease. The pie charts categorise daily activities according to these two domains. Explanations of recorded activities, which emerged through interviews, are also detailed in the results.

#### 2.4.2 Temporal analysis and reasons for movement

Data were analysed thematically in three main stages: coding, categorising and identifying themes (Braun and Clarke, 2006). Interview transcripts and all field notes were coded by hand by the first author. This was an iterative process using both *a priori* concepts based on Prothero's mobility categories, as well as emergent concepts. Deductive codes were refined, and codes that emerged from the data were added and recorded in a codebook. Codes were independently reviewed by an additional researcher. The first author then grouped these codes into categories and themes. Quotations from verbatim transcripts were selected to illustrate these themes.

## 3 Results

### 3.1 Population mobility in the Bijagós islands

Our findings revealed that movement within islands, between islands, and from islands to the mainland was a common feature of island life. Out of 50 respondents, 48 had spent one or more days away from their island of residence during the last 12 months.

Gender and age were key factors determining mobility. Women play an important role in society, which some study respondents associated with the matriarchal elements of Bijagós culture. Many respondents - both men and women - mentioned that Bijogo women work more than men. One man explained:

*In Bijogo culture, women are more powerful than men. Women are the ones who build houses; she is the one who buys clothes for the man. Until this day, Bijogo women keep the men alive.* – KII 1, Bubaque

Women have traditionally been head of the household and take on more responsibility than men. Their duties primarily involve domestic work, caregiving for infants and foraging for materials to build roofs. However, men stated that they are increasingly responsible for constructing houses. Men's roles typically focus on fishing and farming. While both men and women of reproductive age travelled, they did not frequently move in family units. Women were often accompanied by their young children, and men travelled alone or in groups – mirroring the general division of labour. Elders moved much less, often due to reduced physical mobility.

**Fig. 1:**
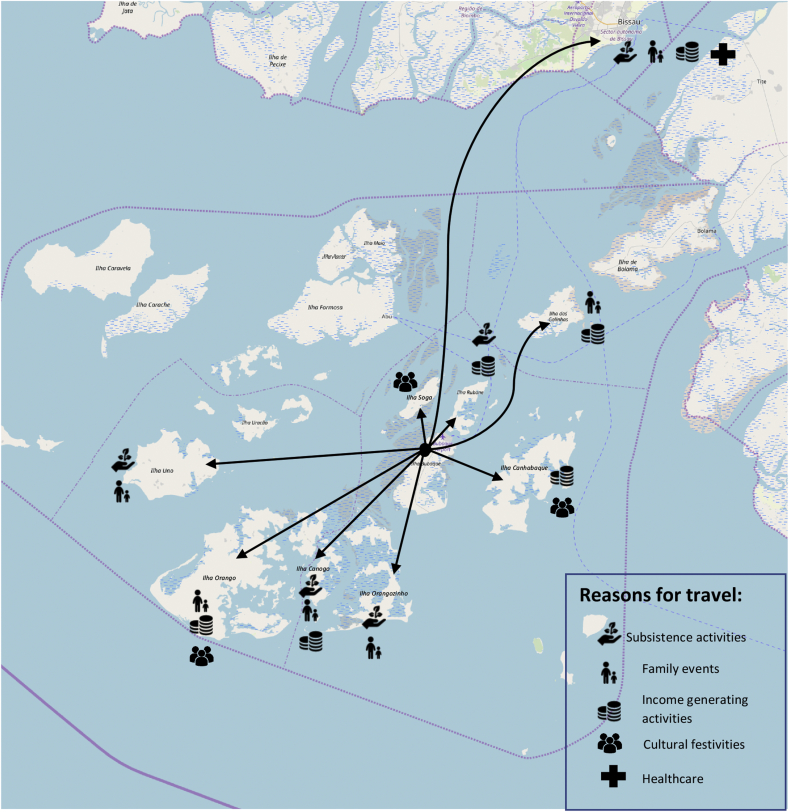
Reasons for and direction of movement away from Bubaque in the past 12 months.

Reasons for travel can be grouped in five broad categories (see Fig. 1): for subsistence (e.g. farming or fishing), economic activities (e.g. exchanging goods to generate income), family events (e.g. visiting relatives or attending a funeral), health reasons (e.g. seeking medical care as a hospital), or cultural festivities (e.g. participating in ceremonies). While study respondents often provided a primary reason for travel, these categories were not mutually exclusive. For example, people who spent time on another island to participate in subsistent farming activities also visited relatives.

**Fig. 2:**
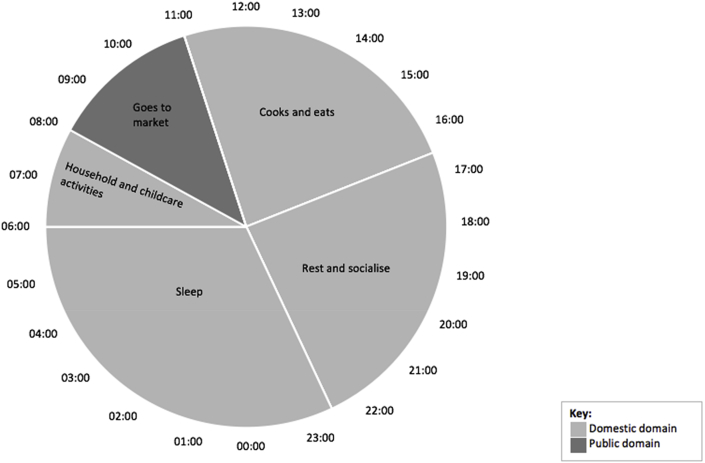
Women's daily activities.

Respondents in Bubaque often visited other islands, particularly Rubane, Soga, Orango and Uno (see Fig. 1). In contrast, residents of Canhabaque travelled to Bubaque or Bissau more than to other islands, likely due to more direct transport options available. Many commented that population movement was common on all islands of the archipelago. Some felt that these patterns had changed over time:

*Bijogo people used to travel mainly between islands. They rarely left the islands. Now, Bijogos are very mobile. You will find them in every city on the mainland.* – KII 1, Bubaque

### 3.2 Temporal nature of and reasons for population movement

Although movement was frequent throughout the year, respondents reported slightly more travel during the calmer weather of the dry season (mid-November to mid-May) when the sea was safer. As it will be explored in the following sections, different activities involved different types of movements.

#### 3.2.1 Daily mobility

Exploring key activities and how these relate to daily movements is important for understanding disease transmission dynamics. For many respondents, daily life in the Bijagós usually followed a similar pattern, with some variation depending on the season or location. In general, women spent more time at home or in the immediate surroundings (the domestic domain) than men. Their primary responsibilities were collecting water, doing domestic work (cooking, cleaning and childcare) and making and selling products. In Bubaque, women in villages and in the town visited the market in the morning, where they were in contact with many other women to buy produce and socialise. Women in villages travelled to the market on foot or hailed a motorcar. Most women returned home to continue domestic duties, although some stayed at the market to sell products. Due to limited trading in Canhabaque, these daily interactions away from home were irregular.

**Fig. 3:**
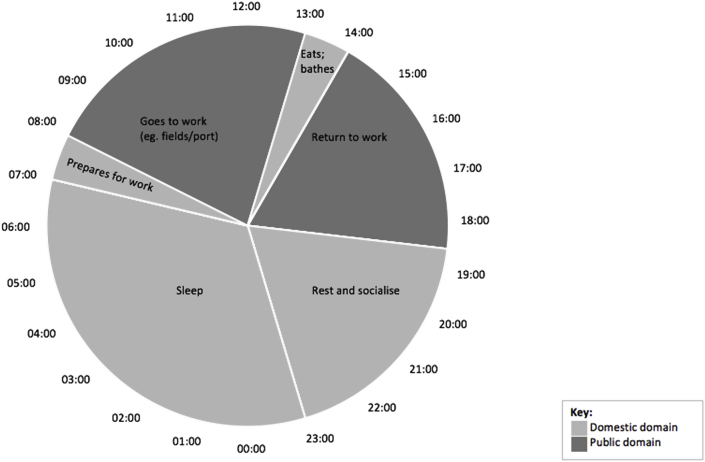
Men's daily activities.

Except for two men employed in administrative government roles, all interviewees worked in the informal sector, primarily as fishermen or in agriculture, or often a mixture of the two. Men often left home early in the morning to begin work (see Fig. 3), whether that consisted of fishing, preparing nets or boats, or working in the forest or fields. These activities took place in different environments and were usually walking distance from their home. Men often returned home briefly in the afternoon to eat with the family and bathe. Most went back to work after, although some stayed to rest and socialise with friends.

In the rainy season (mid-May to mid-November), these activities may take place in different locations. For example, many women spent the whole day in the fields rather than at home, continuing with their usual domestic duties (e.g. cooking) as well as agricultural work.

In addition to the activities highlighted in Fig. 2, Fig. 3, people may also make day trips to visit health clinics when needed or see family in another village or in the town, which often aligns with weekends or school holidays (see Table 1). Interview respondents commented that due to limited transport between islands, most daily activities only involves intra-island movement.

**Table 1:** Types of mobility in the Bijagós and reasons for travel (adapted from Prothero, 1977).

Space	Types of mobility				
	Daily	Short-term periodic (>24 h, <1 month)	Medium-term periodic (>1 month, <12 months)	Seasonal (>24 h, <12 months, relating to season)	Long-term (>12 months)
Intra-island	Collecting water; Fishing; Buying/selling products; Agriculture; Informal work; Family visits; Health reasons	Entertainment; Family visits	Fanado; Family visits	Agriculture (rice, cashew, peanut); Tourism	Education; Work; Traditional duties
Inter-island	–	Fishing; Buying/selling products; Health reasons; Family visits;	Fanado; Entertainment; Buying/selling products; Collecting natural resources; Family visits	Agriculture (rice, cashew, peanut); Tourism; Construction work (eg. building houses/wells); Festivities and holidays (eg. Christmas, Easter, Carnival)	Marriage; Work; Education; Retirement
Island-mainland	–	Boat work; Family visits; Buying/selling products	Boat work; Family visits; Buying/selling products	Agriculture (rice, cashew, peanut)	Marriage; Work; Education; Retirement

#### 3.2.2 Periodic mobility

Periodic mobility can happen at any time of the year. Table 1 highlights some of the key reasons provided by participants for short-term movement that lasts between 24 h and one month, and medium-term movement lasting between 1 and 12 months. Most of these movements occurred in larger groups, although family visits and short-term movement for health reasons sometimes happened individually or in pairs. People often moved for a short period of time when attending cultural festivities. One popular activity is an inter-island football tournament, where young men and women across the archipelago travelled within an island or between islands to the site of the tournament to watch, sleeping in nearby schools or houses for up to one week.

Another significant ceremonial event is the *fanado*, a traditional initiation custom in which age-grade cohorts of men and women separately undergo secret rituals and practices, including circumcision, at different times and places (Cross, 2014). 59% of participants interviewed had either completed a *fanado*, or intended to complete an upcoming *fanado*. *Fanados* take place in a forest, sometimes on the same island, but often on a neighbouring, largely uninhabited island. Respondents in Bubaque reported to have recently travelled to Soga for one week to assist with festivities for the end of a *fanad*o. In Canhabaque, one interviewee explained how the site of each village's *fanado* is different:

*Every village is independent and has a sacred area for the circumcision ceremonies. For example, the port where you arrived is the sacred area. Next year, this place will no longer serve as a port for the inhabitants because of the circumcision ceremonies planned for next year.* – KII 7, Canhabaque

*Fanado* ceremonies are very secretive, and little is known about the specific activities that take place. One participant explained that cohorts of men spend up to three months in the forest where they learn to ‘become a man’. On most islands, men who return from the *fanado* ceremony revert to their normal activities of daily life. However, in Canhabaque, a long-held tradition is still in place where after *fanado,* men enter a period of isolation (*camabé)* that lasts six to seven years. During this time, they live together as a group and are restricted from interacting with any Bijogo women except their blood relatives. During the first year of *camabé*, men must not travel to other islands, but after this period they are able to move freely. One male participant commented on the importance of *fanado* for gaining knowledge and accessing new locations:

*To be more integrated it's important to do the ceremonies. It means you can go to every island. It gives you liberty to move around. Bijog*o*s have lots of secrets.* – IDI 25, Canhabaque

In addition to such events, fishing, trading and collecting natural materials were amongst the most common reasons noted for periodic travel. Fishing is practised throughout the year, although it is more difficult in the rainy season when the weather is less predictable. Inter-island travel involves groups of fishermen who seek out new fishing grounds, and may stay on other islands for several nights at a time.

Women often travelled between islands to collect natural materials, including charcoal, firewood, and reeds for making roofs. These trips occur periodically when the need for materials arise, and often last between one and three months. Women reported travelling with their young babies or primary-school aged children, and staying with relatives or friends on other islands, such as on Rubane or Orango.

Although periodic mobility is most frequent between islands, participants in Bubaque commented that trips to and from the mainland have become more common due to increased trade and transport links with the continent. Men and women travel from Bubaque to trade goods in mainland Bissau, which may take several days to several weeks. Men who work on boats and ferries also frequently move between Bubaque and the mainland numerous times a week. Many participants reported that they had received guests overnight in their home within the last month, but also highlighted the irregular nature of movement:

*There is no specific period when they come. They come all seasons, but they do not stay for long. For example, if they come on a Friday, they leave on the Sunday. Others come for a week; others spend one night and then continue to Bissau.* – KII 2, Bubaque

#### 3.2.3 Seasonal mobility

The dry and wet seasons host different types of activities, requiring travel within or between islands, and to the mainland (see Table 1). The cashew campaign takes place from April–June during the dry season and involves women travelling, often with small children, to other islands or to the mainland for a period of several months. Their destination is dependent on the quality of the cashew crop. Those working in the tourist industry also travel more during the dry season when hotels open for business. Men who work in construction can only build during the dry season, and often travel within and between islands constructing houses, wells or boats during these months. Holidays and festivals including Christmas, Easter and Carnival also encourage many people to travel to other islands or to the mainland for several days to celebrate with family and friends.

Travel in the rainy season is largely for agricultural reasons. Some villages in Bubaque and Canhabaque own rice fields nearby or on other islands. For about six months, from around June–December, whole villages may relocate as a unit to live near fields on their island or on another (sometimes uninhabited) island. They build cabins next to the fields, which results in different social structures and sleeping arrangements. In one temporary settlement by fields in Bubaque, a woman explained that in the village, everyone in her family has their own bed, whereas in the settlement they all slept in one room and shared beds. Cabins near the fields were also positioned differently to the village, resulting in new neighbours and different social relationships forming during the rainy season.

In temporary settlements, daily activities resembled those conducted at home. In the rainy season, both men and women spent large proportions of the day in the fields. Participants reported difficulties accessing drinking water on more remote islands, sometimes relying on assistance from tourist resorts.

In the past, families used to stay together in their temporary settlements during the rainy season. Now, older children return home to their villages when the school year starts and their parents stay by the fields. One participant explained:

*In the past teachers were moving with the students. For example, if the people cultivated land in Rubane, they would build schools and grow rice for the teachers. Now it is not the case as children from other villages come learn in Bijante. So we can't displace the school like in the past. The school remains here, the students come back to the village during school term. -* KII 4, Bubaque

#### 3.2.4 Long-term mobility

In comparison to those who move daily, periodically or seasonally, relatively few participants had moved long-term. Most long-term intra-island mobility is either due to marriage or carrying out traditional duties, such as becoming king of another village (see Table 1). One participant explained that while such movements used to occur in family groups, relatives are now more dispersed:

*In the past, when someone was crowned, he moved with his whole family to the village where he had become King. Over time, this practice has changed little by little. Currently, the King moves alone with maybe a wife and some grandchildren. For example, I have children in school; they do not want to move to a different village.* – KII 7, Canhabaque

Participants who had relocated to another island for more than one year often did so for marriage or work. Those who migrated to or from the mainland said this was for marriage or retirement. Some respondents who had moved to the islands from the mainland spoke of eventual return when they become an elder.

Many different ethnic groups have now emigrated to Bubaque, and some participants had at least one parent from a different ethnic group. By contrast, Canhabaque has attracted a narrower spectrum of ethnic groups. One participant explained:

*There are two ethnic groups who regularly come to Canhabaque. The Pepels share the Bijogo culture with regard to palm tree harvests. And then the Fulani who come here to trade.* - KII 7, Canhabaque

In addition, another participant described a trend of migration from Canhabaque to the mainland:

*Curiously, today, Bijogos from Canhabaque travel the most. They are everywhere on the mainland. They are the ones who cut the fruit from the palm trees in many of the mainland villages.* – KII 1, Bubaque

## 4 Discussion

In order to inform future work on mobility and public health, this study set out to understand the temporal and spatial character of mobility and the reasons for movement within and between islands, and between the archipelago and the mainland, through the specific lens of the Bijogo experience. By showing, for example, that demographic factors such as gender and age affect mobility in different ways and for different reasons, we argue that for its potential public health implications to be appreciated and acted upon, a given pattern of human mobility needs to be considered in its specific details rather than just its overall form.

The main types of movement and reasons for travel provided by participants in this study chimes with other research conducted in sub-Saharan Africa. Our findings highlight that short-distance travel was most common for subsistence and livelihood work, which is similar to other African contexts (Bryceson et al., 2003). Flexible and moving lifestyles of the Bijogo people may show striking similarities with other West African peoples such as the Fulbe of Southern Mali, who are able to shift activities easily due to continuous movements. In many contexts, mobility is the norm while sedentary lifestyles may be unusual (De Bruijn and Van Dijk, 2003).

While mobility patterns in other parts of the world have changed significantly in the 21st century, Prothero's typology remains a useful framework to understand how different types of mobility can impact both individual health and public health programmes in this context (Prothero, 1977). The potential impact of the mobility of the Bijogo people on exposure, transmission and control of infectious diseases will be further explored in the following sections.

### 4.1 Impact on disease exposure and transmission

For infectious diseases with short incubation periods, daily mobility and short-term periodic mobility are thought to be more important than medium-term periodic or long-term movement for transmission (Sattenspiel and Powell, 1993, KRAAY et al., 2018). Daily mobility patterns in the Bijagós Islands could impact disease transmission in several ways. Our results highlight how daily mobility and routines differed by gender. Gendered space has been discussed elsewhere in relation to prevalence, incidence and reinfection of diseases (Watts et al., 1998; Camlin et al., 2018). Among the Bijogo people, daily routines for men were not dominated by a single location or structured workplace – for example, if the weather conditions were not optimal for fishing, men spent the day preparing nets at the port or working in the forest. It is plausible that these weather-dependent, temporally unstructured daily routines (Vazquez-Prokopec et al., 2013) could affect infectious disease dynamics more so than temporally structured routines, due to regular exposure of different ecologies and people.

In contrast to men, who spent many hours away from the home each day, women spent a large proportion of time in the domestic domain. Studies in Tanzania have shown that diseases like *Ascaris* infection are predominantly transmitted in the domestic domain, whereas others such as hookworm may be transmitted in the public domain (Cairncross et al., 1996). These two types of transmission require different interventions. Transmission in the domestic domain can be interrupted through changes in behaviour, whereas transmission in the public domain may require public investment and infrastructure. These domains are relevant to understanding daily mobility and the potential spread of infectious diseases in the Bijagós.

Periodic and seasonal mobility also often varied by gender and by age. Except when whole villages relocated for agricultural purposes, men and women rarely travelled together. Instead, women often travelled with their young children. Research in other African countries has identified women and children as a consistent traveller group that often moves relatively short distances for family reasons (Marshall et al., 2016). This traveller group can significantly contribute towards the spatial spread of malaria due to children having high parasite prevalence and transporting these parasites home (Marshall et al., 2016). Information on key traveller groups can help shape effective disease control and elimination strategies, and should be explored in greater detail in the Bijagós Islands.

It is also important to consider the impact that travel has on access to basic services and how this could impact health (Bartram and Cairncross, 2010). In emergency contexts, temporary resettlement, overcrowding and poor access to safe water and sanitation are all risk factors for infectious disease transmission (Connolly et al., 2004). Similarly in the Bijagós, seasonal agricultural travel can increase environmental and social risk factors. Communities residing in overcrowded temporary settlements with poor water, sanitation and hygiene may be at risk of acquiring infections like *chlamydia trachomatis*, which causes active trachoma (Last et al., 2014).

### 4.2 Impact on public health programmes

In comparison to commonly casual or opportunistic daily and periodic travel, seasonal mobility in the Bijagós tended to be more predictable, with villages moving to different areas for cultivating and harvesting. Understanding these patterns of movement is important for tailoring and increasing the reach of public health interventions (Watson, 2015). While communities that relocate to uninhabited islands for part of the year might not be exposed to new sources of infections, they will typically come into contact with health services less frequently than on settled islands, making them harder to reach through disease control programmes (Smith and Whittaker, 2014). For example, seasonal mobility can deny large fractions of a population access to medications through missing mass drug administration (MDA) events or vaccination campaigns. For many NTDs endemic to the Bijagós archipelago, MDA is the recommended strategy for control or elimination (Webster et al., 2014). Similarly, MDA is increasingly being considered as part of malaria elimination strategies (Newby et al., 2015). Anticipating patterns of mobility can enable programmes and policies to be more effective, such as delivering MDA during seasons where populations are relatively more sedentary (DIAL et al., 2014), extending the duration of campaigns, or running campaigns in occasionally inhabited areas. These strategies could be particularly effective on the Bijagós Islands, where most interventions are community based and coverage remains a challenge (Last et al., 2017). Without knowledge of local movements and disparate risk environments, population mobility can threaten disease elimination efforts (Burkot and Ichimori, 2002).

While there are potential negative impacts of mobility on health, including exposure to diseases through transmission routes identified in Prothero's typology, it is important to acknowledge that these assumptions have not yet been studied in the Bijagós Islands. Research on other islands highlights how mobility may not be detrimental to public health, as it is often assumed. In Zanzibar, short periodic travel between the island and mainland Tanzania was predicted to have played a role in the spread of malaria, but using mobile phone data to track movements, researchers found that travel up to five days had not significantly impacted malaria transmission at a public health level (Tatem et al., 2009). Using these tools and collaborating with community advocates could be one way to accurately map mobility and disease transmission dynamics in more detail.

Our findings highlight that the reasons for population mobility in the Bijagós Islands are multifaceted and complex, and vary significantly at least by gender, age and traveller group. Understanding reasons for movement matters because different types of mobility require tailored disease control strategies (Peeters Grietens et al., 2015; Guyant et al., 2015). Smith and Whittaker have recommended addressing mobility as a system. Mobile populations should not be assumed to be at risk, but mobility should rather be seen as a system involving many demographic groups in different places (Smith and Whittaker, 2014). They argue that this systems approach would improve programmers’ abilities to access populations through community engagement and social networks. Participation is thought to be integral to disease elimination efforts. In the Vanuatu archipelago, a high degree of community participation was associated with the success of a malaria eradication programme, which consisted of short-term mass drug administration and sustained vector control (Kaneko et al., 2000). Understanding local cultural customs, such as *fanado* in the Bijagós, is also key to designing and delivering culturally sensitive and time-appropriate interventions. Adopting a systems approach to mobility in the Bijagós Islands would help understand the phenomenon as part of island life.

### 4.3 Limitations

This study had several limitations. The presence of a non-local, female researcher may have impacted participants’ responses, particularly in rural areas where foreigners are not regularly seen. In addition, the lack of Kriol-English translators available meant a male translator was present during interviews and focus groups with women, which could have also biased the discussion. Several participants did not speak Kriol, and another layer of translation could have meant some meaning was lost through multiple translations.

While detailed notetaking aimed to ensure that data were analysed sufficiently, there is potential that some information was omitted due to the limited number of transcripts produced. As the results presented are based on data collected in Bubaque and Canhabaque only, they may not be generalisable to other islands in the Bijagós.

## 5 Conclusion

To our knowledge, this is the only study to explore population mobility in the Bijagós archipelago. Understanding population movement, as we have attempted to do here, can prompt future work on the impact of different types of mobility on disease transmission and control programmes. In future studies, more attention should be placed on understanding the role of human behaviour in disease transmission in island contexts. In order to address endemic diseases in the archipelago, studies could explore water, sanitation and hygiene-related behavioural risk factors for infectious diseases to shed light on behavioural patterns throughout the process of population mobility. Understanding what drives behaviour around disease prevention, care-seeking and treatment can also contribute towards elimination efforts. Further research on diseases endemic to the islands must consider mobility as more than a risk factor by engaging and empowering communities in the mitigation or elimination of disease.
